# *De novo* Creation and Assessment of a Prognostic Fat-Age-Inflammation Index “FAIN” in Patients With Cancer: A Multicenter Cohort Study

**DOI:** 10.3389/fnut.2022.860285

**Published:** 2022-04-13

**Authors:** Liangyu Yin, Chunhua Song, Jiuwei Cui, Xin Lin, Na Li, Yang Fan, Ling Zhang, Jie Liu, Feifei Chong, Chang Wang, Tingting Liang, Xiangliang Liu, Li Deng, Mei Yang, Jiami Yu, Xiaojie Wang, Xing Liu, Shoumei Yang, Zheng Zuo, Kaitao Yuan, Miao Yu, Minghua Cong, Zengning Li, Min Weng, Qinghua Yao, Pingping Jia, Suyi Li, Zengqing Guo, Wei Li, Hanping Shi, Hongxia Xu

**Affiliations:** ^1^Department of Clinical Nutrition, Daping Hospital, Army Medical University (Third Military Medical University), Chongqing, China; ^2^Institute of Hepatopancreatobiliary Surgery, Southwest Hospital, Army Medical University (Third Military Medical University), Chongqing, China; ^3^Department of Epidemiology, College of Public Health, Zhengzhou University, Zhengzhou, China; ^4^Cancer Center, The First Hospital, Jilin University, Changchun, China; ^5^Department of Medical Oncology, Fujian Cancer Hospital, Fujian Medical University Cancer Hospital, Fuzhou, China; ^6^Department of Nutrition and Metabolism of Oncology, The First Affiliated Hospital of University of Science and Technology of China (Anhui Provincial Cancer Hospital), Hefei, China; ^7^Center of Gastrointestinal Surgery, The First Affiliated Hospital of Sun Yat-sen University, Guangzhou, China; ^8^Department of Comprehensive Oncology, National Cancer Center or Cancer Hospital, Chinese Academy of Medical Sciences and Peking Union Medical College, Beijing, China; ^9^Department of Clinical Nutrition, The First Hospital of Hebei Medical University, Shijiazhuang, China; ^10^Department of Clinical Nutrition, The First Affiliated Hospital of Kunming Medical University, Kunming, China; ^11^Department of Integrated Chinese and Western Medicine, Cancer Hospital of the University of Chinese Academy of Science (Zhejiang Cancer Hospital), Hangzhou, China; ^12^Department of Gastrointestinal Surgery and Department of Clinical Nutrition, Beijing Shijitan Hospital, Capital Medical University, Beijing, China

**Keywords:** cancer, malnutrition, mortality, fat mass, inflammation

## Abstract

**Background and Aims:**

Malnutrition is highly prevalent and is related to multiple impaired clinical outcomes in cancer patients. This study aimed to *de novo* create an objective, nutrition-related index specially for prognostic purposes in oncology populations.

**Methods:**

We performed a multicenter cohort study including 14,134 cancer patients. The prognostic impact for each baseline characteristic was estimated by calculating Harrell's C-index. The optimal parameters reflecting the nutritional and inflammatory impact on patients' overall survival were selected to develop the fat-age-inflammation (FAIN) index. The associations of the FAIN with the nutritional status, physical performance, quality of life, short-term outcomes and mortality of patients were comprehensively evaluated. Independent external validation was performed to further assess the prognostic value of the FAIN.

**Results:**

The study enrolled 7,468 men and 6,666 women with a median age of 57 years and a median follow-up of 42 months. The FAIN index was defined as: (triceps skinfold thickness + albumin) / [age + 5 × (neutrophil count/lymphocyte count)]. There were significant associations of the FAIN with the nutritional status, physical performance, quality of life and short-term outcomes. The FAIN also showed better discrimination performance than the Nutritional Risk Index, the Prognostic Nutritional Index and the Controlling Nutritional Status index (all *P* < 0.05). In multivariable-adjusted models, the FAIN was independently associated with a reduced death hazard both as a continuous variable (HR = 0.57, 95%CI = 0.47–0.68) and per one standard deviation (HR = 0.83, 95%CI = 0.78–0.88). External validation in a multicenter lung cancer cohort (*n* = 227) further confirmed the prognostic value of the FAIN.

**Conclusions:**

This study created and assessed the prognostic FAIN index, which might act as a feasible option to monitor the nutritional status and help develop intervention strategies to optimize the survival outcomes of cancer patients.

## Introduction

Cancer is a huge threat to human health, with an estimated 19.3 million global incident cases and almost 10.0 million deaths annually (1). Despite the recent introduction of new treatment options (2, 3), the poor prognosis of many cancers remains largely unchanged, and the number of new cases is predicted to increase significantly in the foreseeable future (1, 4). Therefore, novel diagnostic, therapeutic and management strategies have been continuously sought, and multimodal cancer care is being emphasized in current oncology practice (5, 6).

Oncology patients frequently experience reduced food intake, weight loss, physical inactivity, metabolic changes and systemic inflammation, which have been ascribed to the chronic consumptive nature of the malignancy itself and/or the side effects of various anti-cancer therapies (7, 8). Thus, they are at particularly high risk for malnutrition compared to other patient groups (9). Additionally, cancer-related malnutrition is often linked to other nutrition status-related conditions such as cachexia and sarcopenia (5, 10–12). These conditions can independently or jointly lead to an impaired quality of life (QOL) (13–15), reduced treatment tolerance (16), increased postoperative complications (17), delayed rehabilitation of organ function (18) and a shortened overall survival (7, 19, 20). Previous studies have estimated that 10–20% of cancer deaths can be attributed to malnutrition rather than the cancer itself (21, 22). However, malnutrition is often underestimated (23), misclassified (24), or left untreated (25) in oncology populations. To address these challenges, the European Society of Clinical Nutrition and Metabolism (ESPEN) recommends in its guidelines that all cancer patients should be evaluated regularly for the risk or presence of malnutrition to guide subsequent intervention strategies (5, 6).

Of the validated approaches used to screen for the risk or assess the severity of malnutrition, the Nutritional Risk Screening 2002 (NRS2002) (26) and the Patient-Generated Subjective Global Assessment (PG-SGA) (27) are the most widely used tools in Chinese oncology patients (15, 19, 28). The Global Leadership Initiative on Malnutrition (GLIM) (11), a set of ESPEN-endorsed guidelines aiming to unify the diagnosis of malnutrition in patients with a wide spectrum of diseases, have also been garnering increasing interest from the nutrition society (7, 8, 14, 15, 17, 19, 20, 28). In addition to these questionnaire- or expert opinion-based tools, several nutrition-related indices have also been implemented to assess the nutritional status of patients, such as the Nutritional Risk Index (NRI) (29), the Controlling Nutritional Status (CONUT) index (30) and the Prognostic Nutritional Index (PNI) (31). These scoring indices were derived from objective laboratory blood tests with/without anthropometric parameters, which have shown significant prognostic value in oncology populations (31–33). However, to our knowledge, although previous studies indicated that cancer patients can have different malnutrition phenotypes, including different anthropometric parameters compared to other patient groups (34, 35), there is not yet an objective, prognosis-oriented, nutrition-related and simple-to-obtain index that is designed specifically for oncology populations.

In the present study conducted in a large-scale, multicenter oncology cohort, we created a prognostic fat-age-inflammation (FAIN) index using a data-driven, outcome-oriented algorithm. We then compared the prognostic performance of the FAIN with five existing scoring systems and comprehensively investigated the associations of the FAIN with other patient characteristics, including the nutritional status, physical performance, QOL and short-term outcomes. Finally, we analyzed the associations of the FAIN, as both a continuous and categorical variable, with cancer mortality.

## Methods

### Population and Design

This was a nationwide, multicenter cohort study. All patients were derived from the Investigation on Nutrition Status and its Clinical Outcome of Common Cancers (INSCOC) project of China which was registered online at https://www.chictr.org.cn (ID: ChiCTR1800020329). The full design of the INSCOC project has been described previously (36) and the detailed inclusion and exclusion criteria are shown in Supplementary Table 1. For the present study, we included 14,908 patients aged over 18 years who were diagnosed with cancer and/or were hospitalized for anti-cancer treatment from November 2011 to April 2019 at multiple centers in four geographical regions (east, south, west and north) of China. After excluding 509 patients with non-solid malignancies and 265 patients with an unclear pathological diagnosis, we finally included 14,134 patients with 17 types of cancer as the study population (Supplementary Figure 1). An independent cohort including 355 esophageal cancer patients diagnosed from December 2014 to November 2019 (not included in the INSCOC project) in our institution was used as the validation set to evaluate the prognostic performance of the FAIN. The study was approved by the Ethics Committees of all participating institutions and all data was analyzed anonymously. All participants in the study provided written consent for the scientific use of their data and the principles of the Declaration of Helsinki were followed.

### Data Acquisition

The following information was collected at baseline within 48 h upon admission by a project-trained researcher via a face-to-face interview or physical examination: age, sex, smoking (active tobacco smoker before admission), alcohol drinking (once a week or more frequent alcohol consumption in the past 1 year, regardless of amount), tea consumption (once a week or more frequent tea consumption in the past 1 year, regardless of amount), comorbidities, height, weight, body mass index (BMI), mid-arm circumference (MAC, non-dominant arm), triceps skinfold thickness (TSF, non-dominant arm), handgrip strength (HGS, non-dominant hand), mid-arm muscle circumference (MAMC), calf circumference (CC, left calf), unintentional weight loss within and beyond 6 months, the NRS2002 score (≥ 3 indicating nutritional risk) (26), the PG-SGA score (27), the Karnofsky Performance Status (KPS) score (37) and the European Organization for Research and Treatment of Cancer QLQ-C30 score (QLQ-C30) (38).

In the present study, the BMI was also categorized as underweight (<18.5 kg/m^2^); normal (18.5 to <24 kg/m^2^), overweight (24 to <28 kg/m^2^), or obese (≥28 kg/m^2^) according to the Chinese recommendation (39). The detailed approaches and instruments used to obtain the anthropometric information (height, weight, BMI, MAC, TSF, HGS, MAMC, CC and weight loss) have been described previously (40), and are also shown in Supplementary Table 2. The gastrointestinal symptoms within the PG-SGA scale were extracted and analyzed independently. For the QLQ-C30, the global QOL scale was used in the present study, with a higher score indicating a better overall QOL.

The disease and treatment information, including the cancer site, clinical stage, differentiation grade, anticancer therapies used, serum indices, length of hospital stay, presence of intensive care unit stay, length of hospitalization, cost and thirty-day death were retrospectively retrieved from electronic medical records. Serum indices were all measured at the clinical laboratories of the participating institutions using fasting blood samples drawn upon admission.

### Follow-Up and Main Outcome

Patients were followed annually after enrollment via telephone or face-to-face interviews to obtain the survival information. The all-cause mortality was the main outcome of the present study, and the overall survival time was calculated as the time interval (months) between the first admission and the patient's date of death, the date of the last valid follow-up, or April 2020.

### Creation of the Fat-Age-Inflammation (FAIN) Index

A data-driven, outcome-oriented approach was used to create an index reflecting the nutritional and inflammatory impact on the patients' overall survival. First, Harrell's C-index was calculated to assess the prognostic impact of each baseline parameter. Then, the TSF (mm) (20), age (years), neutrophil to lymphocyte ratio (NLR, as an inflammatory marker, same unit for the neutrophils and lymphocytes, such as number/L) (15) and the serum albumin (g/L, as an inflammatory and prognostic marker) (41) were manually selected to develop the FAIN index since they showed the highest C-index within their respective categories. The prototypic definition of the FAIN was: (TSF + albumin)/(age + NLR). To maximize the prognostic value, the optimal formula of the FAIN was explored by multiplying each component with different coefficients (the other three parameters remained unchanged during the tuning of one parameter) and the corresponding C-index was observed. The FAIN index was finally determined to be: (TSF + albumin) / [age + 5 × (neutrophil count/lymphocyte count)].

### Statistical Analysis

Continuous data are shown as the medians [interquartile range] and were compared using Wilcoxon's rank-sum test. Categorical data were expressed as numbers (percentage) and compared using a Chi-squared test. The two-variable correlation was examined using a Spearman's rank correlation test. The baseline NRI, PNI and CONUT indices were also calculated to compare their prognostic value with the FAIN according to the following approaches: NRI = (1.519 × serum albumin, g/L) + (41.7 × present weight / usual weight); PNI = 10*serum albumin (g/dl) + 0.005*total lymphocyte count (mm^3^); CONUT includes the serum albumin level, total lymphocyte counts and serum total cholesterol level. The detailed scoring method of the CONUT has been described previously (31).

A restricted cubic spline was used to flexibly analyze the potential non-linear associations of the continuous FAIN index with survival. The potential non-linearity was tested using a likelihood ratio test with *P* < 0.05 indicating a non-linear relationship. We also categorized the continuous FAIN as a dichotomous variable to define the low and high groups using the median value and the optimal stratification (OS)-defined threshold. The OS method selects the threshold for a continuous factor by maximizing the between-group log-rank statistic for the overall survival (42). We also categorized the FAIN in tertiles, quartiles and quintiles to partially minimize the limitations associated with the variable dichotomization. The associations between the FAIN categories and survival was evaluated using Kaplan-Meier curves and log-rank tests. Multivariable-adjusted Cox proportional hazards models were used and hazard ratios (HR) with 95% confidence intervals (95%CIs) were calculated to estimate the association between the FAIN and mortality. We used the Schoenfeld individual test and Kaplan-Meier curves to statistically and visually estimate the proportional hazards assumption for each covariate adjusted (Schoenfeld test *P* > 0.05 indicates that the proportional hazards assumption is satisfied). The linearity assumption between covariates and outcome was confirmed by the Martingale residual plots.

Incremental models with increasing numbers of covariates were created. A dual-direction stepwise method based on the Bayesian Information Criterion (BIC) was used to help select the significant covariates. Model 1 was an unadjusted crude model. Model 2 was adjusted for the age at baseline. Model 3 was adjusted for age, sex and the BIC-screened independent predictors, including the tumor stage, radical surgery, curative chemotherapy, serum prealbumin level, HGS, the NRS2002 score, length of hospital stay and cancer type. Model 4 was adjusted for all variables in Model 3, plus the calf circumference, PG-SGA score, KPS score and the global QOL score.

Sensitivity analyses were performed to test the robustness of the multivariate Cox regression models by excluding the patients who died within the first 3 (Model 5), 6 (Model 6) and 12 months (Model 7), respectively. Multiplicative interactions were tested by adjusting the cross-product terms. Those covariates showing a statistically significant multiplicative interaction (*P* < 0.05) were defined as potential effect modifiers and subgroup analyses were performed in different strata of these variables to evaluate the modification of the associations observed in the overall population. The proportional hazards assumption and linearity assumption were also confirmed for the Cox regression models obtained through stratification using the approaches described above. All tests were two-sided, and *P* < 0.05 was regarded as statistically significant. All analyses were performed using R (version 3.6.3, http://www.rproject.org).

## Results

### Cohort Overview and Derivation of the FAIN

The study included 7,468 men and 6,666 women with a mean age of 57 years. The tumors were most frequently located in the lung (22.9%), colorectum (15.7%), breast (15.6%), stomach (10.6%), esophagus (8.8%), and nasopharynx (7.9%). The predominant clinical stages were III (39.9%) and IV (24.0%). There were 3,266 (23.1%) underweight patients, 7,396 (52.3%) patients with a normal weight, 2,761 (19.5%) overweight patients, and 711 (5.0%) obese patients. There were 3,241 deaths among 14,134 patients during a median follow-up time of 42 months. The patient age (C-index = 0.583, 95%CI = 0.573–0.593), albumin (C-index = 0.595, 95%CI = 0.585–0.605), NLR (C-index = 0.580, 95%CI = 0.570–0.590), and TSF (C-index = 0.589, 95%CI = 0.579–0.599) showed the highest prognostic value among the various baseline data (reflecting the demographic, inflammatory and anthropometric/nutritional dimensions, Table 1), so these were incorporated to develop the FAIN index. A coefficient screen showed that the FAIN had a maximal C-index when the NLR value was multiplied by 5 (Supplementary Table 3). Thus, the formula to generate the FAIN was determined to be: [TSF (mm) + albumin (g/L)]/[age (years) + 5 × (neutrophil count/lymphocyte count)] and the FAIN index was calculated for each patient using the baseline data.

**Table 1:** Baseline information and corresponding Harrell's C-index.

**Characteristics**	**Overall (*n* = 14,134)**	**C-index (95%CI)**
**Demographic information**		
Age, years	57.7 [49.0, 64.9]^a^	0.583 (0.573–0.593)
Sex, male, *n* (%)	7,468 (52.8)	0.575 (0.567–0.583)
Smoking, yes, *n* (%)	5,949 (42.1)	0.567 (0.557–0.577)
Alcohol drinking, yes, *n* (%)	2,802 (19.8)	0.535 (0.527–0.543)
Tea consumption, yes, *n* (%)	3,315 (23.5)	0.515 (0.507–0.523)
**Comorbidities**, ***n*** **(%)**		
Hypertension	2,428 (17.2)	0.507 (0.499–0.515)
Diabetes	1,123 (7.9)	0.508 (0.502–0.514)
Chronic hepatitis	668 (4.7)	0.504 (0.500–0.508)
Coronary heart disease	637 (4.5)	0.501 (0.497–0.505)
Chronic biliary disease	581 (4.1)	0.502 (0.498–0.506)
Anemia	470 (3.3)	0.501 (0.497–0.505)
**Laboratory indices**		
Total protein, g/L	67.9 [63.0, 72.4]	0.527 (0.517–0.537)
Prealbumin, mg/L	210.0 [167.0, 256.0]	0.574 (0.564–0.584)
Albumin, g/L	39.4 [35.8, 42.6]	0.595 (0.585–0.605)
Transferrin, g/L	2.3 [1.9, 2.7]	0.538 (0.528–0.548)
Urea nitrogen, mmol/L	5.0 [4.0, 6.2]	0.492 (0.480–0.504)
Creatinine, mmol/L	65.0 [55.0, 77.0]	0.523 (0.513–0.533)
Total bilirubin, μmol/L	10.7 [7.9, 14.5]	0.516 (0.506–0.526)
Direct bilirubin, μmol/L	2.9 [2.1, 4.0]	0.491 (0.479–0.503)
Alanine transaminase, U/L	18.3 [12.7, 29.0]	0.511 (0.501–0.521)
Aspartate aminotransferase, U/L	21.8 [17.1, 28.7]	0.527 (0.515–0.539)
Cholesterol, mmol/L	4.5 [3.9, 5.2]	0.482 (0.472–0.492)
Glucose, mmol/L	5.3 [4.8, 5.9]	0.504 (0.494–0.514)
Triglycerides, mmol/L	1.2 [0.9, 1.7]	0.490 (0.480–0.500)
High density lipoprotein, mmol/L	1.2 [1.0, 1.4]	0.528 (0.518–0.538)
Low density lipoprotein, mmol/L	2.8 [2.3, 3.4]	0.513 (0.503–0.523)
Hemoglobin, g/L	125.0 [110.0, 137.0]	0.539 (0.529–0.549)
White blood cells, ×10^9^/L	6.1 [4.8, 7.9]	0.538 (0.528–0.548)
Red blood cells, ×10^12^/L	4.2 [3.8, 4.6]	0.546 (0.536–0.556)
Platelets, ×10^9^/L	222.0 [171.0, 281.0]	0.508 (0.496–0.520)
Neutrophils, ×10^9^/L	3.8 [2.7, 5.5]	0.553 (0.543–0.563)
Lymphocytes, ×10^9^/L	1.5 [1.1, 1.9]	0.445 (0.435–0.455)
**Systemic inflammation**		
C-reactive protein, mg/L	3.4 [1.7, 15.0]	0.575 (0.565–0.585)
Neutrophil to lymphocyte ratio	2.5 [1.7, 4.2]	0.580 (0.570–0.590)
**Anthropometric parameters**		
Height, cm	163.0 [158.0, 169.0]	0.545 (0.535–0.555)
Weight, kg	60.0 [53.0, 67.5]	0.529 (0.519–0.539)
Body mass index, kg/m^2^	21.3 [18.8, 23.9]	0.529 (0.519–0.539)
**Body mass index category, kg/m**^**2**^, ***n*** **(%)**		0.523 (0.513–0.533)
Underweight (<18.5)	3,266 (23.1)	
Normal (18.5 to <24)	7,396 (52.3)	
Overweight (24 to <28)	2,761 (19.5)	
Obese (≥28)	711 (5.0)	
Mid-arm circumference, cm	26.5 [24.5, 28.5]	0.546 (0.536–0.556)
Triceps skinfold thickness, mm	16.0 [11.0, 22.0]	0.589 (0.579–0.599)
Handgrip strength, kg	23.8 [17.8, 30.8]	0.506 (0.496–0.516)
Mid-arm muscle circumference, cm	21.3 [19.4, 23.2]	0.519 (0.509–0.529)
Calf circumference, cm	33.0 [31.0, 35.5]	0.554 (0.544–0.564)
Weight loss within 6 months, %	0.0 [0.0, 3.6]	0.549 (0.539–0.559)
Weight loss beyond 6 months, %	2.2 [0.0, 8.2]	0.545 (0.535–0.555)
**Disease and treatment**		
**Cancer site**, ***n*** **(%)**		0.679 (0.671–0.687)
Lung	3,231 (22.9)	
Colorectum	2,215 (15.7)	
Breast	2,204 (15.6)	
Stomach	1,497 (10.6)	
Esophagus	1,241 (8.8)	
Nasopharynx	1,111 (7.9)	
Cervix	635 (4.5)	
Liver	447 (3.2)	
Lymphoma	398 (2.8)	
Ovary	343 (2.4)	
Pancreas	206 (1.5)	
Bladder	148 (1.0)	
Endometrium	141 (1.0)	
Prostate	140 (1.0)	
Biliary	92 (0.7)	
Brain	70 (0.5)	
Gastric stroma	15 (0.1)	
**Clinical stage**, ***n*** **(%)**		0.622 (0.612–0.632)
I	1,863 (13.2)	
II	3,229 (22.8)	
III	5,645 (39.9)	
IV	3,397 (24.0)	
**Differentiation grade**, ***n*** **(%)**		0.540 (0.530–0.550)
Well	954 (6.7)	
Moderate	6,302 (44.6)	
Poor	5,181 (36.7)	
None-differentiated	1,697 (12.0)	
**Anticancer therapies**, ***n*** **(%)**		
Radical surgery	5,524 (39.1)	0.571 (0.563–0.579)
Curative radiotherapy	877 (6.2)	0.512 (0.506–0.518)
Curative chemotherapy	2,507 (17.7)	0.555 (0.547–0.563)
Preoperative neoadjuvant chemotherapy	633 (4.5)	0.509 (0.505–0.513)
Postoperative adjuvant chemotherapy	3,032 (21.5)	0.537 (0.531–0.543)
Chemotherapy for metastasis	1,133 (8.0)	0.524 (0.518–0.530)
Other anticancer therapy	2,778 (19.7)	0.534 (0.526–0.542)
**Scales**		
NRS2002 score, continuous	2.0 [1.0, 4.0]	0.578 (0.568–0.588)
NRS2002, ≥3, *n* (%)	6,268 (44.3)	0.561 (0.551–0.571)
PG-SGA score, continuous	4.0 [2.0, 8.0]	0.593 (0.583–0.603)
**PG-SGA category**, ***n*** **(%)**		0.590 (0.580–0.600)
0–1	3,460 (24.5)	
2–3	3,254 (23.0)	
4–8	4,397 (31.1)	
≥9	3,023 (21.4)	
KPS score	90.0 [80.0, 100.0]	0.570 (0.560–0.580)
Global QOL score	66.7 [50.0, 83.3]	0.556 (0.546–0.566)

*CI, confidence interval; NRS2002, the Nutritional Risk Screening 2002; PG-SGA, the Patient-Generated Subjective Global Assessment; KPS, the Karnofsky Performance Status; QOL, quality of life score by the European Organization for Research and Treatment of Cancer (EORTC) QLQ-C30 scale.*

a *Median [interquartile range], all such values*.

### The FAIN and Patient Characteristics

The baseline patient characteristics, as stratified by the median-dichotomized FAIN, are presented in Table 2. Compared to the FAIN low group, the FAIN high group was associated with a higher value/rate of total protein, pre-albumin, albumin, transferrin, alanine transaminase, cholesterol, triglycerides, high density lipoprotein, low density lipoprotein, hemoglobin, red blood cells, platelets, lymphocytes, weight, BMI, MAC, TSF, HGS, CC, radical surgery, postoperative adjuvant chemotherapy, KPS score, global QOL score, NRI score, and PNI score, and was associated with a lower value/rate of age, male sex, smoking, alcohol drinking, tea consumption, hypertension, diabetes, coronary heart disease, chronic biliary disease, anemia, urea nitrogen, creatinine, total bilirubin, direct bilirubin, glucose, white blood cells, neutrophils, C-reactive protein, NLR, height, MAMC, weight loss within and beyond 6 months, curative radiotherapy, curative chemotherapy, other anticancer therapy, NRS2002 score, PG-SGA score, gastrointestinal symptoms (no appetite, nausea, vomiting, constipation, dry mouth, things taste funny or have no taste, dysphagia, feel full quickly, abdominal pain and other symptoms) and the CONUT score. As was expected, the cancer types, clinical stage and differentiation grade were also different between the low and high FAIN groups. Additionally, a univariate analysis on the short-term outcomes also showed that a higher FAIN was associated with a shorter length of hospital stay, fewer incidents of an intensive care unit stay, lower costs during hospitalization and lower rates of thirty-day mortality (all *P* < 0.05).

**Table 2:** Associations between the patient characteristics and median-dichotomized FAIN^a^.

**Characteristics**	**FAIN low (*n* = 7,067)**	**FAIN high (*n* = 7,067)**	***P***
**Demographic information**			
Age, years	63.4 [57.6, 69.4]^b^	50.8 [44.1, 57.9]	<0.001
Age, ≥65 years, *n* (%)	2,976 (42.1)	491 (6.9)	<0.001
Sex, male, *n* (%)	4,716 (66.7)	2,752 (38.9)	<0.001
Smoking, yes, *n* (%)	3,765 (53.3)	2,184 (30.9)	<0.001
Alcohol drinking, yes, *n* (%)	1,745 (24.7)	1,057 (15.0)	<0.001
Tea consumption, yes, *n* (%)	1,889 (26.7)	1,426 (20.2)	<0.001
**Comorbidities**, ***n*** **(%)**			
Hypertension	1,460 (20.7)	968 (13.7)	<0.001
Diabetes	695 (9.8)	428 (6.1)	<0.001
Chronic hepatitis	346 (4.9)	322 (4.6)	0.362
Coronary heart disease	427 (6.0)	210 (3.0)	<0.001
Chronic biliary disease	317 (4.5)	264 (3.7)	0.028
Anemia	264 (3.7)	206 (2.9)	0.007
**Laboratory indices**			
Total protein, g/L	65.5 [60.2, 70.3]	69.9 [65.7, 73.8]	<0.001
Prealbumin, mg/L	190.0 [139.0, 232.4]	230.0 [195.0, 270.0]	<0.001
Albumin, g/L	36.8 [33.2, 40.1]	41.5 [38.8, 44.1]	<0.001
Transferrin, g/L	2.2 [1.8, 2.6]	2.4 [2.1, 2.8]	<0.001
Urea nitrogen, mmol/L	5.2 [4.1, 6.4]	4.8 [3.9, 5.9]	<0.001
Creatinine, mmol/L	66.9 [55.9, 79.0]	64.0 [55.0, 75.0]	<0.001
Total bilirubin, μmol/L	10.9 [8.0, 14.9]	10.5 [7.9, 14.1]	<0.001
Direct bilirubin, μmol/L	3.0 [2.1, 4.5]	2.7 [2.0, 3.7]	<0.001
Alanine transaminase, U/L	17.7 [12.0, 28.1]	19.0 [13.0, 29.6]	<0.001
Aspartate aminotransferase, U/L	22.0 [17.0, 29.8]	21.5 [17.8, 28.0]	0.105
Cholesterol, mmol/L	4.4 [3.8, 5.1]	4.6 [4.0, 5.3]	<0.001
Glucose, mmol/L	5.3 [4.8, 6.1]	5.2 [4.8, 5.8]	<0.001
Triglycerides, mmol/L	1.1 [0.9, 1.6]	1.3 [1.0, 1.8]	<0.001
High density lipoprotein, mmol/L	1.2 [1.0, 1.4]	1.2 [1.0, 1.4]	<0.001
Low density lipoprotein, mmol/L	2.8 [2.3, 3.3]	2.9 [2.4, 3.4]	<0.001
Hemoglobin, g/L	120.0 [105.0, 134.0]	128.0 [116.0, 140.0]	<0.001
White blood cells, ×10^9^/L	6.7 [5.2, 8.9]	5.7 [4.5, 7.1]	<0.001
Red blood cells, ×10^12^/L	4.1 [3.6, 4.5]	4.3 [4.0, 4.7]	<0.001
Platelets, ×10^9^/L	218.0 [165.0, 282.0]	226.0 [178.0, 279.0]	<0.001
Neutrophils, ×10^9^/L	4.6 [3.2, 6.7]	3.3 [2.4, 4.4]	<0.001
Lymphocytes, ×10^9^/L	1.3 [0.9, 1.7]	1.7 [1.3, 2.1]	<0.001
**Systemic inflammation**			
C-reactive protein, mg/L	6.4 [3.0, 30.7]	3.1 [1.0, 6.0]	<0.001
Neutrophil to lymphocyte ratio	3.7 [2.3, 6.2]	1.9 [1.4, 2.7]	<0.001
**Anthropometric parameters**			
Height, cm	165.0 [158.0, 170.0]	161.0 [157.0, 168.0]	<0.001
Weight, kg	58.0 [51.0, 65.0]	61.8 [55.0, 69.5]	<0.001
Body mass index, kg/m^2^	20.5 [18.1, 23.0]	21.9 [19.5, 24.6]	<0.001
**Body mass index category, kg/m**^**2**^, ***n*** **(%)**			<0.001
Underweight (<18.5)	2,131 (30.2)	1,135 (16.1)	
Normal (18.5 to <24)	3,553 (50.3)	3,843 (54.4)	
Overweight (24 to <28)	1,160 (16.4)	1,601 (22.7)	
Obese (≥28)	223 (3.2)	488 (6.9)	
Mid-arm circumference, cm	26.0 [23.5, 28.0]	27.5 [25.3, 29.5]	<0.001
Triceps skinfold thickness, mm	12.0 [9.0, 16.0]	20.0 [15.0, 25.0]	<0.001
Handgrip strength, kg	23.5 [17.1, 30.0]	24.0 [18.5, 31.7]	<0.001
Mid-arm muscle circumference, cm	21.5 [19.6, 23.5]	21.1 [19.1, 23.0]	<0.001
Calf circumference, cm	32.0 [30.0, 34.5]	34.0 [32.0, 36.0]	<0.001
Weight loss within 6 months, %	0.0 [0.0, 4.8]	0.0 [0.0, 2.0]	<0.001
Weight loss beyond 6 months, %	3.8 [0.0, 10.0]	0.9 [0.0, 6.2]	<0.001
**Disease and treatment**			
**Cancer site**, ***n*** **(%)**			<0.001
Lung	1,946 (27.5)	1,285 (18.2)	
Colorectum	1,203 (17.0)	1,012 (14.3)	
Breast	352 (5.0)	1,852 (26.2)	
Stomach	1,023 (14.5)	474 (6.7)	
Esophagus	964 (13.6)	277 (3.9)	
Nasopharynx	272 (3.8)	839 (11.9)	
Cervix	216 (3.1)	419 (5.9)	
Liver	270 (3.8)	177 (2.5)	
Lymphoma	192 (2.7)	206 (2.9)	
Ovary	136 (1.9)	207 (2.9)	
Pancreas	138 (2.0)	68 (1.0)	
Bladder	104 (1.5)	44 (0.6)	
Endometrium	39 (0.6)	102 (1.4)	
Prostate	114 (1.6)	26 (0.4)	
Biliary	71 (1.0)	21 (0.3)	
Brain	17 (0.2)	53 (0.7)	
Gastric stroma	10 (0.1)	5 (0.1)	
**Clinical stage**, ***n*** **(%)**			<0.001
I	768 (10.9)	1,095 (15.5)	
II	1,674 (23.7)	1,555 (22.0)	
III	2,554 (36.1)	3,091 (43.7)	
IV	2,071 (29.3)	1,326 (18.8)	
**Differentiation grade**, ***n*** **(%)**			<0.001
Well	499 (7.1)	455 (6.4)	
Moderate	3,282 (46.4)	3,020 (42.7)	
Poor	2,733 (38.7)	2,448 (34.6)	
None-differentiated	553 (7.8)	1,144 (16.2)	
**Anticancer therapies**, ***n*** **(%)**			
Radical surgery	2,549 (36.1)	2,975 (42.1)	<0.001
Curative radiotherapy	567 (8.0)	310 (4.4)	<0.001
Curative chemotherapy	1,375 (19.5)	1,132 (16.0)	<0.001
Preoperative neoadjuvant chemo	170 (2.4)	463 (6.6)	<0.001
Postoperative adjuvant chemo	1,157 (16.4)	1,875 (26.5)	<0.001
Chemotherapy for metastasis	562 (8.0)	571 (8.1)	0.804
Other anticancer therapy	1,579 (22.3)	1,199 (17.0)	<0.001
**Scales**			
NRS2002 score, continuous	4.0 [2.0, 4.0]	1.0 [1.0, 4.0]	<0.001
NRS2002, ≥3, *n* (%)	4,131 (58.5)	2,137 (30.2)	<0.001
PG-SGA score, continuous	6.0 [2.0, 10.0]	2.0 [1.0, 5.0]	<0.001
**PG-SGA category**, ***n*** **(%)**			<0.001
0–1	829 (11.7)	2,631 (37.2)	
2–3	1,539 (21.8)	1,715 (24.3)	
4–8	2,430 (34.4)	1,967 (27.8)	
≥9	2,269 (32.1)	754 (10.7)	
KPS score	90.0 [80.0, 90.0]	90.0 [90.0, 100.0]	<0.001
Global QOL	66.7 [50.0, 75.0]	66.7 [58.3, 83.3]	<0.001
**Gastrointestinal symptoms**, ***n*** **(%)**			
No appetite	1,359 (19.2)	736 (10.4)	<0.001
Nausea	600 (8.5)	392 (5.5)	<0.001
Vomiting	419 (5.9)	182 (2.6)	<0.001
Mouth sores	72 (1.0)	61 (0.9)	0.384
Constipation	549 (7.8)	348 (4.9)	<0.001
Diarrhea	227 (3.2)	199 (2.8)	0.184
Dry mouth	474 (6.7)	369 (5.2)	<0.001
Things taste funny or have no taste	416 (5.9)	238 (3.4)	<0.001
Smells bother me	158 (2.2)	85 (1.2)	<0.001
Dysphagia	640 (9.1)	183 (2.6)	<0.001
Feel full quickly	471 (6.7)	315 (4.5)	<0.001
Abdominal pain	542 (7.7)	356 (5.0)	<0.001
Other	209 (3.0)	81 (1.1)	<0.001
NRI	95.6 [89.1, 101.4]	103.9 [99.1, 108.4]	<0.001
PNI	43.6 [39.1, 47.6]	50.2 [46.6, 53.8]	<0.001
CONUT	3.0 [1.0, 4.0]	1.0 [0.0, 2.0]	<0.001
**Short-term outcomes**			
Length of hospital stay, days	13.0 [8.0, 20.0]	12.0 [7.0, 19.0]	<0.001
Intensive care unit stay, yes, *n* (%)	1,317 (18.6)	1,054 (14.9)	<0.001
Cost, 10,000 RMB yuan	2.5 [1.2, 5.7]	1.9 [1.0, 3.7]	<0.001
Thirty-day mortality, *n* (%)	177 (2.5)	49 (0.7)	<0.001

*FAIN, the fat-age-inflammation index; NRS2002, the Nutritional Risk Screening 2002; PG-SGA, the Patient-Generated Subjective Global Assessment; KPS, the Karnofsky Performance Status; QOL, quality of life score by the European Organization for Research and Treatment of Cancer (EORTC) QLQ-C30 scale; NRI, the Nutritional Risk Index; PNI, the Prognostic Nutritional Index; CONUT, the Controlling Nutritional Status index.*

a *Median of FAIN = 0.77; FAIN high, ≥0.77; FAIN low, <0.77.*

b *Median [interquartile range], all such values*.

### Correlations

**Figure 1:**
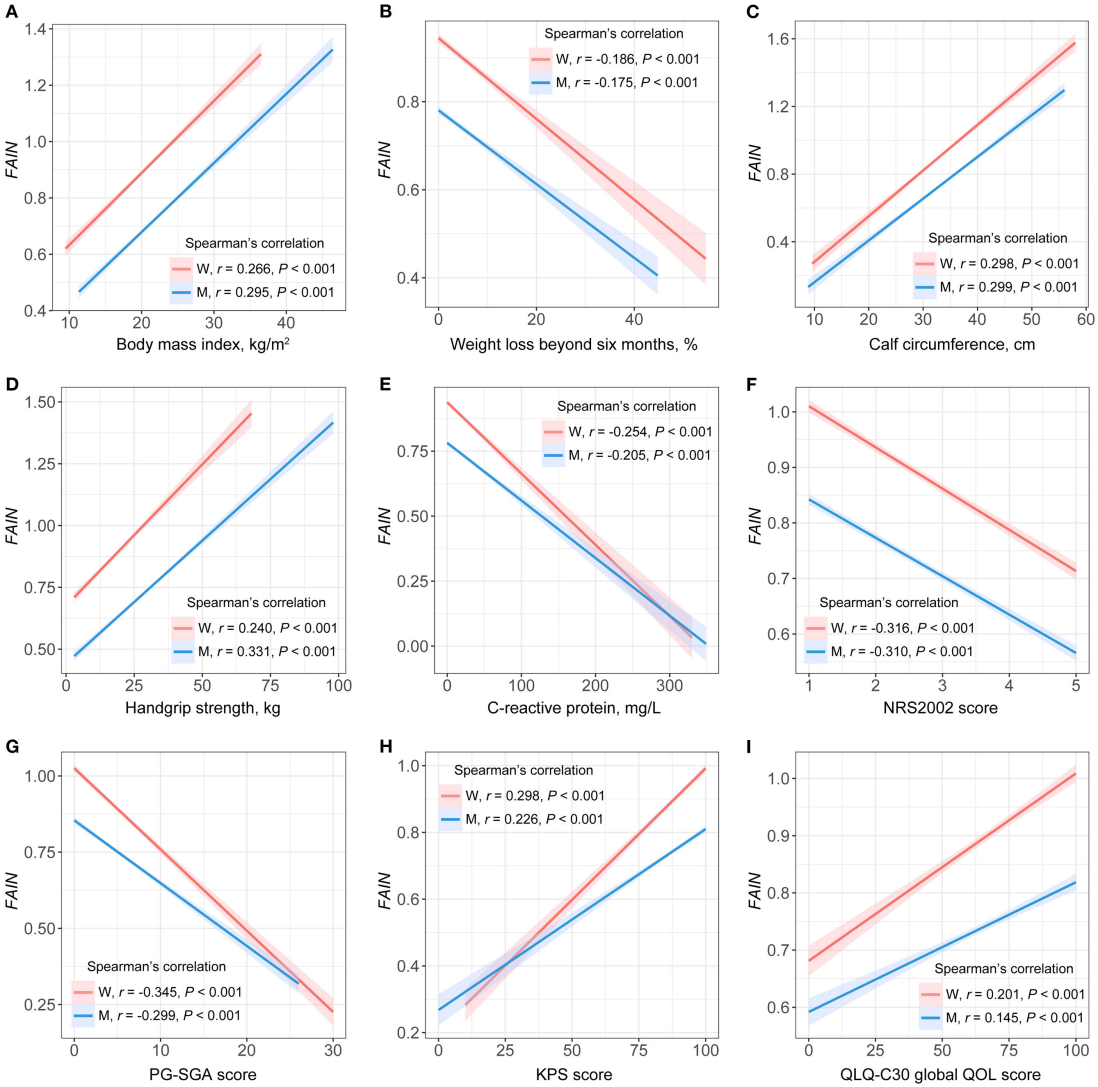
Spearman's rank correlations between the fat-age-inflammation (FAIN) index and patient characteristics. W, women; M, men; NRS2002, the Nutritional Risk Screening 2002; PG-SGA, the Patient-Generated Subjective Global Assessment; KPS, the Karnofsky Performance Status score; QOL, quality of life. **(A)** FAIN vs. body mass index. **(B)** FAIN vs. weight loss beyond six months. **(C)** FAIN vs. calf circumference. **(D)** FAIN vs. handgrip strength. **(E)** FAIN vs. C-reactive protein. **(F)** FAIN vs. NRS2002. **(G)** FAIN vs. PG-SGA. **(H)** FAIN vs. KPS score. **(I)** FAIN vs. QLQ-C30 global QOL score.

Sex-specific spearman's rank correlation tests were performed to assess the degree of relevance for the associations of the continuous FAIN with the BMI, weight loss beyond 6 months, CC, HGS, C-reactive protein, NRS2002 score, PG-SGA score, KPS score and global QOL score (Figure 1). The results were similar for both genders, showing a positive correlation between the FAIN and BMI (Figure 1A), CC (Figure 1C), HGS (Figure 1D), KPS score (Figure 1H) and global QOL score (Figure 1I), and a negative correlation between FAIN and weight loss (Figure 1B), C-reactive protein (Figure 1E), NRS2002 score (Figure 1F) and PG-SGA score (Figure 1G, all *P* < 0.05).

### Prognostic Value Compared to Five Existing Systems

The Harrell's C-index of the FAIN was statistically compared to those calculated for the NRI, PNI, CONUT, NRS2002, and PG-SGA. The results showed that the FAIN had the highest prognostic value, with a C-index = 0.634 (95%CI = 0.624–0.644) compared to the NRI (C-index = 0.599, 95%CI = 0.589–0.609, *P* < 0.001), PNI (C-index = 0.595, 95%CI = 0.585–0.605, *P* < 0.001), CONUT (C-index = 0.575, 95%CI = 0.565–0.585, *P* < 0.001), NRS2002 (C-index = 0.578, 95%CI = 0.568–0.587, *P* < 0.001), and PG-SGA (C-index = 0.593, 95%CI = 0.583–0.603, *P* < 0.001).

### Univariate Survival Analysis

**Figure 2:**
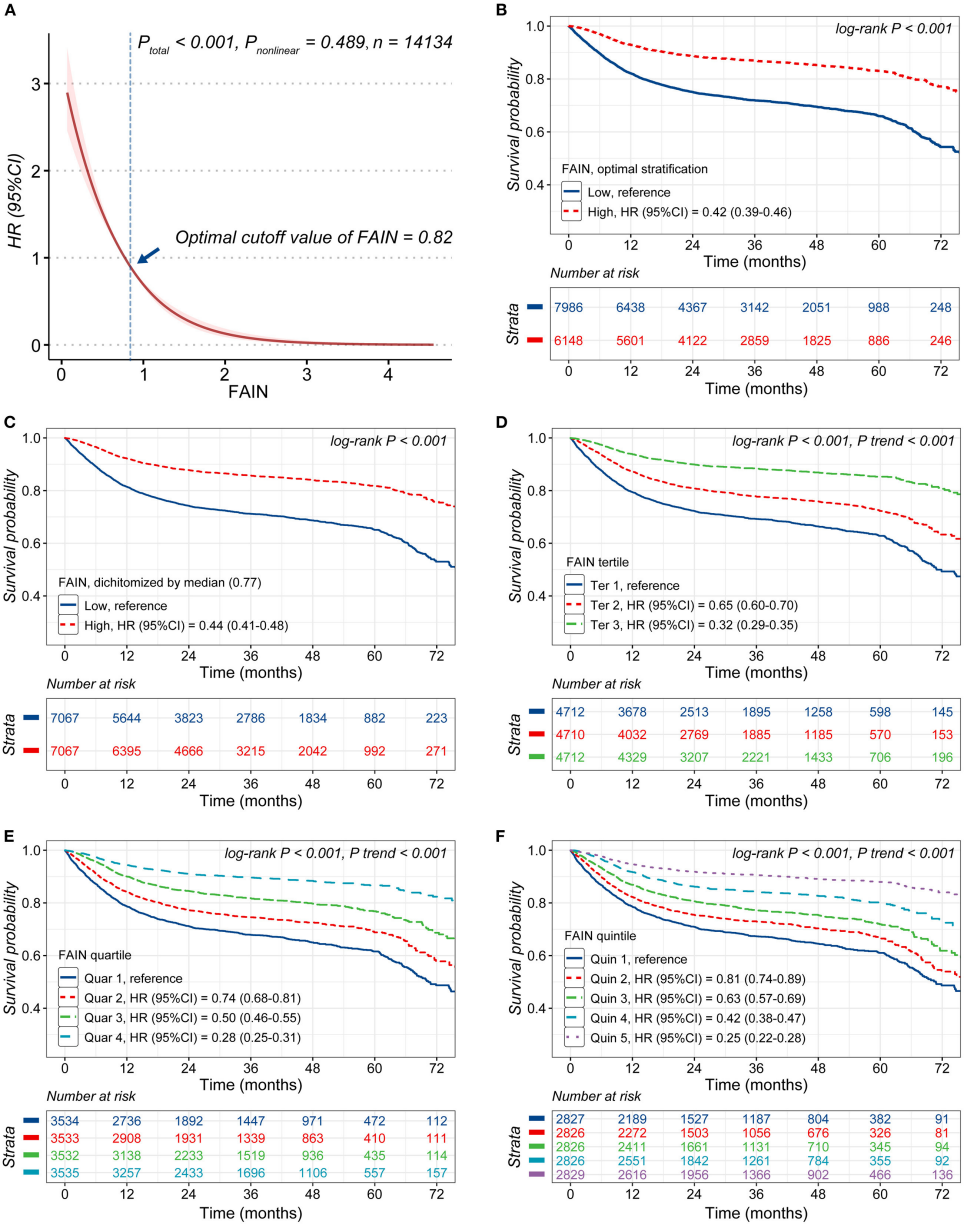
Analysis of the associations of the fat-age-inflammation (FAIN) index with survival. **(A)** Restricted cubic spine (RCS) analysis of the association of the FAIN with survival. **(B)** Kaplan-Meier curves stratified by FAIN (dichotomized using the threshold calculated by optimal stratification). **(C)** Kaplan-Meier curves stratified by the FAIN (dichotomized using the median value). **(D)** Kaplan-Meier curves stratified by FAIN tertiles. **(E)** Kaplan-Meier curves stratified by FAIN quartiles. **(F)** Kaplan-Meier curves stratifi ed by FAIN quintiles.

A restricted cubic spline analysis showed that the continuous FAIN index was associated with a reduced mortality risk (*P* < 0.001) and no significant non-linearity was observed for this relationship (*P* = 0.489). The optimal threshold of the FAIN was 0.82, as determined by the OS (high: ≥0.82; low: <0.82, Figure 2A). Kaplan-Meier curves demonstrated that patients with a higher FAIN had better overall survival than those in the lower groups, regardless of the categorization approach used (all *P* < 0.001). For the FAIN tertiles, quartiles and quintiles, the tests for *P* of the trend indicated that the FAIN was monotonically associated with better overall survival of the patients (all *P* for trend <0.001, Figures 2B–F).

### Multivariable Survival Analysis

The results of the multivariable Cox proportional hazards models on the associations between the FAIN and mortality are shown in Table 3. The FAIN was analyzed as a continuous variable, per each standard deviation and as a categorical variable. In the fully-adjusted model (Model 4), the FAIN was independently associated with a reduced death hazard as both a continuous variable (HR = 0.57, 95%CI = 0.47–0.68) and for separation by one standard deviation (HR = 0.83, 95%CI = 0.78–0.88). Similar results were observed when the FAIN was dichotomized based on the OS (HR = 0.71, 95%CI = 0.64–0.78) or the median value (HR = 0.78, 95%CI = 0.71–0.86). These associations were all sustained when excluding those patients died within the first 3 (Model 5), 6 (Model 6) and 12 months (Model 7), except that the association between the median-dichotomized FAIN and mortality was not sustained in Model 7. This partially supports the use of the OS to generate an outcome-oriented FAIN threshold.

**Table 3:** Multivariable models for the FAIN and overall survival.

	**Overall population, HR (95%CI), cases/events** **=** **14,134/3,241**				**Sensitivity analysis, HR (95%CI)**		
**Models**	**Model 1^**a**^**	**Model 2^**b**^**	**Model 3^**c**^**	**Model 4^**d**^**	**Model 5^**e**^**	**Model 6^**f**^**	**Model 7^**g**^**
FAIN, continuous	0.21 (0.18–0.24)	0.23 (0.19–0.26)	0.51 (0.43–0.61)	0.57 (0.47–0.68)	0.67 (0.55–0.82)	0.67 (0.54–0.84)	0.68 (0.52–0.89)
FAIN, per 1 SD (0.3)	0.59 (0.56–0.62)	0.61 (0.57–0.64)	0.80 (0.75–0.85)	0.83 (0.78–0.88)	0.87 (0.82–0.93)	0.88 (0.81–0.94)	0.88 (0.80–0.96)
FAIN, OS, high vs. low	0.42 (0.39–0.46)	0.47 (0.43–0.52)	0.71 (0.64–0.78)	0.71 (0.64–0.78)	0.71 (0.64–0.78)	0.71 (0.64–0.78)	0.86 (0.74–0.99)
FAIN, high vs. low^h^	0.44 (0.41–0.48)	0.50 (0.46–0.54)	0.74 (0.68–0.81)	0.78 (0.71–0.86)	0.85 (0.77–0.94)	0.88 (0.79–0.98)	0.89 (0.78–1.03)
**FAIN, tertile**							
Tertile 1	Reference	Reference	Reference	Reference	Reference	Reference	Reference
Tertile 2	0.65 (0.60–0.70)	0.67 (0.62–0.72)	0.84 (0.77–0.91)	0.86 (0.79–0.94)	0.92 (0.84–1.01)	0.92 (0.84–1.01)	0.96 (0.84–1.10)
Tertile 3	0.32 (0.29–0.35)	0.35 (0.31–0.39)	0.61 (0.54–0.69)	0.65 (0.58–0.74)	0.73 (0.64–0.84)	0.73 (0.64–0.84)	0.79 (0.65–0.95)
**FAIN, quartile**							
Quartile 1	Reference	Reference	Reference	Reference	Reference	Reference	Reference
Quartile 2	0.74 (0.68–0.81)	0.75 (0.69–0.81)	0.86 (0.79–0.94)	0.88 (0.81–0.97)	0.92 (0.83–1.02)	0.89 (0.79–0.99)	0.89 (0.77–1.03)
Quartile 3	0.50 (0.46–0.55)	0.51 (0.47–0.57)	0.74 (0.66–0.82)	0.78 (0.70–0.87)	0.86 (0.77–0.97)	0.88 (0.77–1.00)	0.89 (0.75–1.05)
Quartile 4	0.28 (0.25–0.31)	0.29 (0.25–0.33)	0.54 (0.47–0.63)	0.59 (0.51–0.68)	0.65 (0.55–0.76)	0.63 (0.53–0.76)	0.68 (0.54–0.85)
**FAIN, quintile**							
Quintile 1	Reference	Reference	Reference	Reference	Reference	Reference	Reference
Quintile 2	0.81 (0.74–0.89)	0.81 (0.74–0.89)	0.89 (0.81–0.98)	0.91 (0.82–1.00)	0.95 (0.85–1.05)	0.92 (0.81–1.03)	0.88 (0.76–1.03)
Quintile 3	0.63 (0.57–0.69)	0.63 (0.57–0.70)	0.82 (0.73–0.91)	0.85 (0.76–0.95)	0.91 (0.80–1.02)	0.92 (0.81–1.05)	0.90 (0.76–1.07)
Quintile 4	0.42 (0.38–0.47)	0.43 (0.38–0.48)	0.66 (0.58–0.75)	0.70 (0.62–0.80)	0.81 (0.70–0.93)	0.83 (0.71–0.97)	0.82 (0.67–0.99)
Quintile 5	0.25 (0.22–0.28)	0.25 (0.22–0.30)	0.51 (0.43–0.60)	0.55 (0.46–0.65)	0.61 (0.51–0.73)	0.63 (0.51–0.77)	0.60 (0.46–0.77)

*HR (95%CI), hazard ratio (95% confidence interval); FAIN, the fat-age-inflammation index; SD, standard deviation; OS, optimal stratification.*

a *Model 1 is the unadjusted crude model.*

b *Model 2 is adjusted for the age at baseline (continuous).*

c *Model 3 is adjusted for the age at baseline (continuous), sex (reference = female), tumor stage (reference = I), radical surgery (reference = no), curative chemotherapy (reference = no), pre-albumin (continuous), handgrip strength (continuous), the Nutritional Risk Screening 2002 (reference = <3), length of hospital stay (continuous) and cancer type (reference = lung cancer).*

d *Model 4 is adjusted for all variables in Model 3, plus the calf circumference (continuous), Patient-Generated Subjective Global Assessment score (reference = 0–1), Karnofsky Performance Status score (continuous) and the global quality of life score (continuous).*

e *Model 5 is adjusted for all covariates in Model 4, but excluded the patients who died within the first 3 months after enrollment (cases/events = 13,626/2,734).*

f *Model 6 is adjusted for all covariates in Model 4, but excluded the patients who died within the first 6 months after enrollment (cases/events = 13,125/2,236).*

g *Model 7 is adjusted for all covariates in Model 4, but excluded the patients who died within the first 12 months after enrollment (cases/events = 12,039/1,388).*

h *High, ≥median (0.77); low, < median (0.77).*

The results were also similar when the FAIN was analyzed as tertiles, quartiles or quintiles. Compared to those in the lowest tertile, patients in the highest tertile had a significantly reduced death hazard (Model 4, HR = 0.65, 95%CI = 0.58–0.74). Similar patterns were observed for the highest quartile (Model 4, HR = 0.59, 95%CI = 0.51–0.68, vs. the lowest quartile) and the highest quintile (Model 4, HR = 0.55, 95%CI = 0.46–0.65, vs. the lowest quintile). These associations were all sustained in the sensitivity analysis (Model 5–7) and tests for *P* of the trend showed that the positive associations between the FAIN and overall survival were all “dose-dependent” (all *P* for trend <0.001).

### Interaction and Subgroup Analysis

All covariates were screened for potential interactive effects, and the patient sex, tumor stage, curative chemotherapy, prealbumin, cancer type, PG-SGA category, KPS score and global QOL score showed statistically significant interactions with the FAIN (all *P* < 0.05). The fully-adjusted models were then repeated in different variable strata to study the effect modifications (Table 4). The positive association between the FAIN and overall survival (HR = 0.57, 95%CI = 0.47–0.68, as continuous) was strengthened in female patients (HR = 0.40, 95%CI = 0.30–0.54), those with stage III tumors (HR = 0.49, 95%CI = 0.36–0.67), patients who did not receive curative chemotherapy (HR = 0.51, 95%CI = 0.41–0.64), the higher serum prealbumin group (HR = 0.52, 95%CI = 0.40–0.67), those with lung cancer (HR = 0.47, 95%CI = 0.34–0.66), colorectal cancer (HR = 0.39, 95%CI = 0.23–0.64) and other cancers (HR = 0.53, 95%CI = 0.38–0.73), those in PG-SGA category B (HR = 0.48, 95%CI = 0.38–0.63), with a higher KPS (HR = 0.52, 95%CI = 0.43–0.64) and in the higher global QOL group (HR = 0.39, 95%CI = 0.26–0.58). In contrast, this relationship was attenuated in male patients (HR = 0.70, 95%CI = 0.56–0.88), those with a lower tumor stage (stage I, HR = 0.86, 95%CI = 0.45–1.64; stage II, HR = 0.72, 95%CI = 0.46–1.13), patients who received curative chemotherapy (HR = 0.69, 95%CI = 0.49–0.98), the lower serum prealbumin group (HR = 0.63, 95%CI = 0.48–0.82), those with breast (HR = 0.90, 95%CI = 0.36–2.25) and gastric cancer (HR = 0.97, 95%CI = 0.71–1.34), patients in PG-SGA category A (HR = 0.59, 95%CI = 0.37–0.93) and C (HR = 0.83, 95%CI = 0.61–1.12), with a lower KPS (HR = 0.76, 95%CI = 0.45–1.29) and those in the lower global QOL group (HR = 0.63, 95%CI = 0.51–0.77).

**Table 4:** Multivariable models stratified by factors showing interactive effects with the FAIN.

	**HR (95%CI)**			
**Model**	**Cases/events**	**FAIN, continuous**	**FAIN, per 1 SD (0.3)**	***P*** ^**inter**^
Crude model, overall	14,134/3,241	0.21 (0.18–0.24)	0.59 (0.56–0.62)	-
Fully-adjusted model^a^, ^b^, overall	14,134/3,241	0.57 (0.47–0.68)	0.83 (0.78–0.88)	-
**Sex**				0.002
Female	6,666/1,130	0.40 (0.30–0.54)	0.74 (0.67–0.81)	
Male	7,468/2,111	0.70 (0.56–0.88)	0.89 (0.82–0.96)	
**Tumor stage**				<0.001
I	1,863/216	0.86 (0.45–1.64)	0.95 (0.77–1.18)	
II	3,229/545	0.72 (0.46–1.13)	0.90 (0.77–1.04)	
III	5,645/1,154	0.49 (0.36–0.67)	0.79 (0.71–0.87)	
IV	3,397/1,326	0.57 (0.43–0.76)	0.83 (0.75–0.91)	
**Curative chemotherapy**				0.001
No	11,627/2,386	0.51 (0.41–0.64)	0.80 (0.74–0.86)	
Yes	2,507/855	0.69 (0.49–0.98)	0.88 (0.78–0.99)	
**Pre-albumin, mg/L**				0.006
<200	5,745/1,658	0.63 (0.48–0.82)	0.86 (0.78–0.94)	
≥200	8,389/1,583	0.52 (0.40–0.67)	0.80 (0.73–0.87)	
**Cancer type**				0.044
Lung	3,231/1,225	0.47 (0.34–0.66)	0.78 (0.69–0.87)	
Colorectum	2,215/430	0.39 (0.23–0.64)	0.73 (0.61–0.86)	
Breast	2,204/143	0.90 (0.36–2.25)	0.97 (0.71–1.31)	
Stomach	1,497/450	0.97 (0.71–1.34)	0.99 (0.89–1.10)	
Other	4,987/993	0.53 (0.38–0.73)	0.81 (0.73–0.90)	
**PG–SGA category**				0.005
A (0–1)	3,460/489	0.59 (0.37–0.93)	0.84 (0.72–0.98)	
B (2–8)	7,615/1,757	0.48 (0.38–0.63)	0.78 (0.72–0.85)	
C (≥9)	3,023/995	0.83 (0.61–1.12)	0.94 (0.85–1.04)	
**KPS score**				0.013
<80	1,535/459	0.76 (0.45–1.29)	0.91 (0.76–1.09)	
≥80	12,599/2,782	0.52 (0.43–0.64)	0.80 (0.75–0.86)	
**Global QOL score**				0.004
<80	10,002/2,515	0.63 (0.51–0.77)	0.86 (0.80–0.92)	
≥80	4,132/726	0.39 (0.26–0.58)	0.73 (0.64–0.83)	

*FAIN, the fat-age-inflammation index; HR (95%CI), hazard ratio (95% confidence interval); SD, standard deviation; P^inter^, P for interaction; KPS, the Karnofsky Performance Status score; QOL, quality of life score by the European Organization for Research and Treatment of Cancer (EORTC) QLQ-C30 scale.*

a *Model is adjusted for the age at baseline (continuous), sex (reference = female), tumor stage (reference = I), radical surgery (reference = no), curative chemotherapy (reference = no), pre-albumin (continuous), handgrip strength (continuous), the Nutritional Risk Screening (NRS) 2002 (reference = <3), length of hospital stay (continuous), cancer type (reference = lung cancer), calf circumference (continuous), the Patient-Generated Subjective Global Assessment (PG-SGA) score (reference = 0–1), the KPS score (continuous) and the global QOL score (continuous).*

b *Interaction test, vs. the FAIN: age = 0.097, sex = 0.002, tumor stage <0.001, radical surgery = 0.538, curative chemotherapy = 0.001, pre-albumin = 0.006, handgrip strength = 0.764, NRS2002 = 0.334, length of hospitalization = 0.346, cancer type = 0.044, calf circumference = 0.565, PG-SGA = 0.005, KPS score = 0.013 and global QOL = 0.004*.

### Independent Validation

The prognostic impact of the FAIN was further assessed in an independent multicenter lung cancer cohort (*n* = 227) which was not used for the derivation of the FAIN. The baseline characteristics of the validation cohort are shown in Supplementary Table 4. The Harrell's C-index of the FAIN in the validation cohort was 0.639 (95%CI = 0.586–0.691) which was higher than the NRI (C-index = 0.589, 95%CI = 0.535–0.643, *P* = 0.030), PNI (C-index = 0.591, 95%CI = 0.536–0.647, *P* = 0.031), CONUT (C-index = 0.572, 95%CI = 0.518–0.627, *P* < 0.001), NRS2002 (C-index = 0.554, 95%CI = 0.502–0.607, *P* = 0.003), and PG-SGA (C-index = 0.585, 95%CI = 0.535–0.634, *P* = 0.031). Consistent with the findings in the original dataset, a multivariable Cox regression analysis in the validation cohort also showed that the FAIN was independently associated with a reduced death hazard, both as a continuous variable (HR = 0.21, 95%CI = 0.06–0.76) and when assessed as per one standard deviation (HR = 0.70, 95%CI = 0.53–0.94, Supplementary Table 5).

## Discussion

This was a large-scale, observational cohort study including 14,134 patients with 17 cancers at multiple centers in China. Based on a data-driven, outcome-oriented approach, we developed a new prognostic index, the FAIN, that integrates information on the inflammation and nutrition. To our knowledge, this is the first study to date that proposes such an index specially designed for oncology populations. We demonstrated that this index effectively reflects the nutritional status, physical performance and QOL of the patients, and is associated with the short-term clinical outcomes of patients. We also performed parallel comparisons that indicated that the FAIN index has better discrimination performance to predict cancer mortality than the existing NRI, PNI, CONUT, NRS2002 and PG-SGA systems in the study population. We revealed that the FAIN is independently associated with the death hazard. Importantly, the components used to create the FAIN index were simple to obtain, and the association between the FAIN and mortality is linear-like and robust to time. Additionally, we validated the performance of the FAIN in an independent lung cancer cohort. These findings suggest that the FAIN might act as a feasible, cost-effective option to monitor the nutritional status of patients and help develop intervention strategies to optimize the survival outcomes of cancer patients.

A distinct feature of the FAIN index is the inclusion of a fat mass assessment, which is not included in most existing scoring systems such as the NRI, PNI and CONUT. The PNI and CONUT only consist of serum laboratory indices, while the NRI also considers some anthropometric changes of patients (e.g., weight loss) (31). However, the weight loss parameter is often obtained based on patient-reported usual/historic weights, which is subject to recall bias that can cause instability when calculating the NRI. In contrast, the fat mass assessment (through measurement of the skinfold thickness) is a relatively objective parameter, which was included in the PG-SGA (27), a nutritional assessment tool dedicated to oncology patients which is currently recommended for use in China. A previous study conducted in a large Chinese oncology cohort also indicated that a low TSF was associated with poorer nutritional status and had greater prognostic impact on cancer mortality than other muscle parameters such as the CC and MAMC (40). A lower TSF was also associated with increased death hazard and actually enhanced the prognostic value of the GLIM-diagnosed malnutrition in lung cancer patients (20). Similarly, the positive association between a low TSF and mortality was also reported in patients with cancer cachexia (43) and in terminally ill cancer patients (44). These results are consistent with our observations in the present study and further support the inclusion of the TSF in the FAIN. Additionally, the inclusion of an objective measurement of body fat might partially explain the superior prognostic value of the FAIN compared to the other three scoring systems in cancer patients. However, the impact of the fat mass on cancer mortality can vary based on the cancer type (40). For example, higher adiposity was associated with higher all-cause and cancer-specific mortality in breast cancer patients (45). In the present study, the favorable impact of the FAIN on patient survival was also attenuated in breast cancer patients (Table 4), which might suggest that the FAIN would be of limited use in breast cancer patients. Intriguingly, a recent study conducted in a large dataset has shown that, paradoxically, in patients with HER2-positive advanced breast cancer, a higher BMI was independently associated with improved survival (46). Since we lack the data about HER2 expression in our patients, this possible link cannot be assessed in our study cohort, and future studies with gene test results are needed to clarify the role of the FAIN in greater detail among breast cancer patients. Another related concern is the potential impact of sex difference of TSF on the prognostic performance of the FAIN. To examine this, we calculated sex-specific FAIN thresholds (male < 0.69 or female < 0.82) based on the OS method to defined a low FAIN in an exploratory analysis. However, this leaded to a statistically significant reduction of the Harrell's C-index (0.592 vs. 0.601, *P* = 0.002) compared to the current threshold (<0.82) calculated for the overall study population. Therefore, pragmatically, we used the gender-neutral threshold 0.82 to maximize the prognostic value of the dichotomized FAIN in the present study. Nevertheless, the optimal approach to define a low FAIN should be re-evaluated when the FAIN index is used for non-prognostic purposes in future studies. In an exploratory analysis, we also calculated thresholds for the most prevalent lung cancer (value = 0.83) and colorectal cancer (value = 0.68) based on the OS method. However, limited to the study scope, future studies need to evaluate the prognostic value of these thresholds in specific cancer groups.

The definition of malnutrition is still not possible using a universally-accepted framework (11, 19, 26–28), largely due to the factors including the diversity of indices used for its identification, the different parameter thresholds, racial/disease-specific differences, complicated etiology and even the continuously evolving but inconsistent understanding of this issue (11, 19, 28). Of note, fat mass assessment was not included as a component in the recent GLIM criteria that were proposed for assessing malnutrition (11). However, depletion of the free fat mass is prevalent in cancer patients, especially among those undergoing chemotherapy/radiotherapy or having cancer cachexia (10), and has been correlated with impaired clinical outcomes (47, 48). A recent study conducted in a Chinese lung cancer population also indicated that adding the TSF can help assess nutritional status and enhance the prognostic value of GLIM-defined malnutrition (20). In support of that study, our present findings also suggest that fat mass assessment might be helpful during the assessment of cancer patients for malnutrition. However, since the present study did not consider the use and impact of nutritional intervention, future studies are still needed to explore whether inclusion of a fat mass assessment during the nutritional assessment would help guide the subsequent nutritional intervention in cancer patients.

There are several potential limitations of this study that must be noted. First, we used a data-driven approach to derive the FAIN index, so the associations between the FAIN and cancer mortality might not be generalizable to other populations. Future validation of the FAIN is needed in all types of cancer and in different populations with characteristics different from those of the group where it was developed before being put into routine clinical or research applications. Second, some the associations we observed in the multivariable survival analysis may be explained by reverse causality. However, we performed sensitivity analyses by excluding those patients who died within the first 3, 6, and 12 months, and the results were robust to time, which should help to reduce this probability. Third, unmeasured confounders are possible in all observational studies. However, we comprehensively collected the baseline characteristic of patients and adjusted the covariates based on both statistical and scientific approaches to minimize this possibility. Fourth, since Asian populations have anthropometric differences compared with their Western counterparties (12), the generalizability of the FAIN should be re-evaluated when applied in non-Asian oncology populations. Fifth, we proposed median value and outcome-oriented threshold that transformed the FAIN into a dichotomous variable (low vs. high). However, dichotomizing continuous variables can lead to an reduction of information (49). Although additional statistical approaches (by analyzing the FAIN as continuous, per standard deviation and percentiles) might provide additional insights, future assessment is still required to determine the optimal grouping algorithm/risk intervals of the FAIN to facilitate its clinical use. Sixth, although being inexpensive and simple, TSF was less accurate to measure body fat than those parameters obtained from more advanced technologies such as dual-energy X-ray absorptiometry. Future studies need to assess the certainty of the FAIN index in greater details. Eighth, we did not have data on other treatments (besides anticancer therapies) which might confound the associations we observed in the present study. Ninth, since not all of the continuous variables (such as the weight loss percentage) had normal distribution, we conservatively used non-parametric statistical approaches to test between-group differences despite the large sample size. Parametric method may have better performance for some normally-distributed continuous variables. Future studies need to address the above issues.

In conclusion, this study *de novo* created and assessed a prognostic index, the FAIN, that integrates information on the patient fat mass/nutrition, age and inflammation. This index effectively reflects the nutritional status, physical performance and QOL of oncology patients, and is associated with improved short-term clinical outcomes. The FAIN has better discrimination performance to predict cancer mortality than the existing NRI, PNI, CONUT, NRS2002, and PG-SGA systems. The impact of the FAIN on cancer mortality is linear-like, independent and robust to time. These findings suggest that the FAIN might act as a feasible, simple-to-obtain option to monitor the nutritional status and help develop intervention strategies to optimize the survival outcomes of cancer patients.

## Data Availability Statement

The datasets generated and/or analyzed during the current study are not publicly available to protect patient confidentiality but are available from the corresponding author on reasonable request.

## Ethics Statement

The studies involving human participants were reviewed and approved by the Ethics Committee of Daping Hospital. The patients/participants provided their written informed consent to participate in this study.

## Author Contributions

LY, WL, HS, and HX designed the study. CS, JC, XinL, NL, YF, LZ, JL, FC, CW, TL, XiaL, LD, MYa, JY, XW, XingL, SY, ZZ, KY, MYu, MC, ZL, MW, QY, PJ, SL, and ZG recruited participants and collected data. LY conducted the study, analyzed the data, and drafted the manuscript. All authors read and approved the final manuscript.

## Funding

This work was supported by the Chongqing Municipal Science Committee, Health Commission Joint Research Project (2019QNXM008, YF), the National Key Research and Development Program (2017YFC1309200, HS), and the Clinical Science Foundation of the Daping Hospital (2014YLC08, HX).

## Conflict of Interest

The authors declare that the research was conducted in the absence of any commercial or financial relationships that could be construed as a potential conflict of interest.

## Publisher's Note

All claims expressed in this article are solely those of the authors and do not necessarily represent those of their affiliated organizations, or those of the publisher, the editors and the reviewers. Any product that may be evaluated in this article, or claim that may be made by its manufacturer, is not guaranteed or endorsed by the publisher.

## Supplementary Material

Supplementary Figure 1 A flow chart of the patient inclusion.

Supplementary Table 1 Inclusion and exclusion criteria for the INSCOC project.

Supplementary Table 2 Approaches and instruments used to obtain the anthropometric information in the present study.

Supplementary Table 3 The different coefficients of each parameter in the fat-age-inflammation (FAIN) index and the corresponding C-index.

Supplementary Table 4 Baseline characteristics of the validation lung cancer cohort.

Supplementary Table 5 Multivariable models for the fat-age-inflammation (FAIN) index and overall survival in the validation cohort.

## Abbreviations

FAIN: the fat-age-inflammation index
HR: hazard ratio
CI: confidence interval
QOL: quality of life
ESPEN: the European Society of Clinical Nutrition and Metabolism
NRS2002: the Nutritional Risk Screening 2002
PG-SGA: the Patient-Generated Subjective Global Assessment
GLIM: the Global Leadership Initiative on Malnutrition
NRI: the Nutritional Risk Index
CONUT: the Controlling Nutritional Status index
PNI: the Prognostic Nutritional Index
INSCOC: the Investigation on Nutrition Status and its Clinical Outcome of Common Cancers project of China
BMI: body mass index
MAC: mid-arm circumference
TSF: triceps skinfold thickness
HGS: hand grip strength
MAMC: mid-arm muscle circumference
CC: calf circumference
KPS: the Karnofsky Performance Status score
QLQ-C30: the European Organization for Research and Treatment of Cancer QLQ-C30 score
NLR: neutrophil to lymphocyte ratio
OS: optimal stratification
BIC: the Bayesian Information Criterion.
