# Supplementary figures and images for: De novo Creation and Assessment of a Prognostic Fat-Age-Inflammation Index “FAIN” in Patients With Cancer: A Multicenter Cohort Study
Source: Front Nutr. 2022 Apr 13;9:860285. doi: 10.3389/fnut.2022.860285 (PMC9043856; doi:10.3389/fnut.2022.860285)

Figure S1

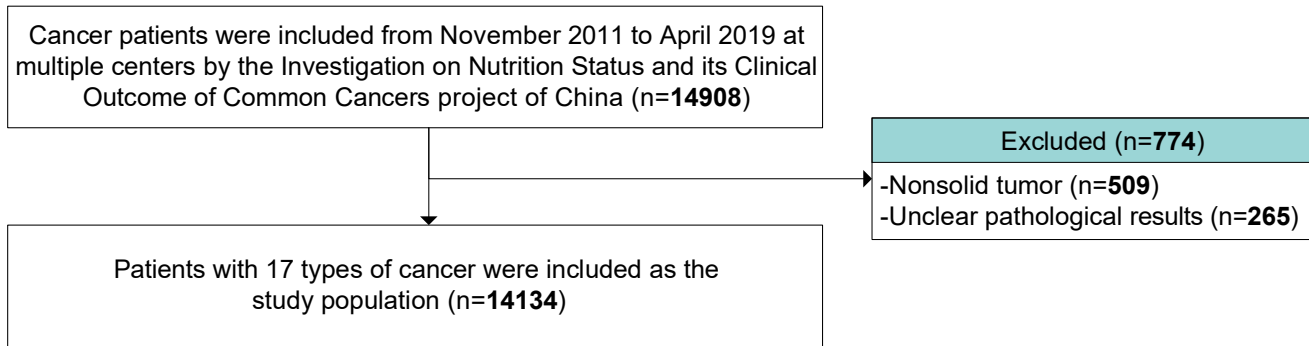

Supplement: Supplementary Figure 1 — A flow chart of the patient inclusion. [file Image_1.PDF]
