# Supplementary material for: De novo Creation and Assessment of a Prognostic Fat-Age-Inflammation Index “FAIN” in Patients With Cancer: A Multicenter Cohort Study
Source: Front Nutr. 2022 Apr 13;9:860285. doi: 10.3389/fnut.2022.860285 (PMC9043856; doi:10.3389/fnut.2022.860285)
Supplement: Supplementary Table 1 — Inclusion and exclusion criteria for the INSCOC project. [file Data_Sheet_1.PDF]

Table S1. Inclusion and exclusion criteria for the INSCOC project

Inclusion criteria	Exclusion criteria
1) Age at least 18 years;<br>2) With length of hospital stay longer than 48 hours;<br>3) Diagnosed with one of the following 18 types of locally or metastatic malignant tumors: lung cancer, gastric cancer, liver cancer, colorectal cancer, breast cancer, esophageal cancer, cervical cancer, endometrial cancer, nasopharyngeal carcinoma, malignant lymphoma, leukemia, pancreatic cancer, ovarian cancer, prostate cancer, bladder cancer, brain tumors, biliary tract malignant tumors and gastrointestinal stromal tumors	1) With organ transplantation;<br>2) Pregnant woman;<br>3) Diagnosed with HIV infection or AIDS;<br>4) Admitted to the ICU at the beginning of recruitment;<br>5) If patients were hospitalized more than two times during the investigation, only the data from the first survey were included

INSCOC, Investigation on Nutrition Status and its Clinical Outcome of Common Cancers (chictr.org.cn: ChiCTR1800020329); HIV, human immunodeficiency virus; AIDS, acquired immunodeficiency syndrome; ICU, intensive care unit.

Table S2. Approaches and instruments used to obtain the anthropometric information in the present study

Parameter	Instrument	Method
Body weight (kg) and height (cm)	Height & weight measuring instrument (Model SH-201, Zhengzhou, China)	The body weight and height of patients were measured using an integrated height and weight measuring instrument in patients wearing light indoor clothing without shoes, to the nearest 0.1 kg and 0.1 cm, respectively, within 48 hours of the first admission.
Body mass index (kg/m^2)	Not applicable	The body mass index was calculated as the weight in kilograms divided by the height in meters squared.
Mid-arm circumference (MAC, non-dominant arm, cm)	Not applicable	The mid-arm circumference (MAC, non-dominant arm) was measured using a flexible and non-elastic tape to the nearest 0.1 cm. The MAC was measured while the patient was in a standing position with the non-dominant arm and the elbow relaxed. The measuring tape was placed around the upper arm at the midpoint perpendicular to the long axis of the upper arm and the value was recorded. When measuring the MAC, the tape was pressed to the skin surface without tight compression.
Calf circumference (CC, left calf, cm)	Not applicable	The calf circumference (CC, left calf) was measured using a flexible and non-elastic tape to the nearest 0.1 cm. The CC was measured while the patient was in a seated position, with the measurement taken on a perpendicular plane to the long axis of the left calf to obtain the maximal value. When measuring the CC, the tape was pressed to the skin surface without tight compression.
Triceps skinfold thickness (TSF, non-dominant arm, mm)	Adipometer (PZJ-01, Jiangsu, China)	The TSF was measured while the patient was in a seated position with shoulders relaxed and arms hanging freely at the sides. The researcher pinched about 2.0 cm of the triceps skinfold and measured it using the adipometer with its jaws held perpendicular to the shaft of the arm.
Mid-arm muscle circumference (MAMC, cm)	Not applicable	The MAMC was calculated as MAC-3.14 × TSF (cm).
Handgrip strength (HGS, kg)	Handgrip dynamometer (CAMRY, Model EH101, Guangdong, China)	Patients were asked to stand comfortably, then to perform three maximal isometric contractions 30s apart using their non-dominant hand. The maximum read for the hand grip strength was recorded.

Table S3. The different coefficients of each parameter in the fat-age-inflammation (FAIN) index and the corresponding C-index

	Harrell's C-index (95%CI)			
Coefficient	Triceps skinfold thickness	Age	Neutrophil to lymphocyte ratio	Albumin
Squared	0.608 (0.598 - 0.618)	0.613 (0.603 - 0.624)	0.630 (0.620 - 0.639)	0.617 (0.607 - 0.627)
1	0.630 (0.620 - 0.640)	0.630 (0.620 - 0.640)	0.630 (0.620 - 0.640)	0.630 (0.620 - 0.640)
2	0.629 (0.619 - 0.639)	0.629 (0.619 - 0.639)	0.632 (0.622 - 0.642)	0.628 (0.618 - 0.638)
3	0.627 (0.617 - 0.637)	0.628 (0.618 - 0.638)	0.633 (0.623 - 0.643)	0.625 (0.615 - 0.635)
4	0.625 (0.615 - 0.635)	0.627 (0.617 - 0.637)	0.634 (0.624 - 0.644)[a]	0.623 (0.613 - 0.633)
5	0.623 (0.613 - 0.633)	0.627 (0.617 - 0.637)	0.634 (0.624 - 0.644)[b]	0.622 (0.612 - 0.632)
6	0.622 (0.612 - 0.632)	0.627 (0.617 - 0.637)	0.634 (0.624 - 0.644)[c]	0.621 (0.611 - 0.631)
7	0.621 (0.611 - 0.631)	0.626 (0.616 - 0.636)	0.633 (0.623 - 0.643)	0.620 (0.610 - 0.630)
8	0.620 (0.610 - 0.630)	0.626 (0.616 - 0.636)	0.633 (0.623 - 0.643)	0.620 (0.610 - 0.630)
9	0.620 (0.610 - 0.630)	0.626 (0.616 - 0.636)	0.632 (0.622 - 0.642)	0.619 (0.609 - 0.629)
10	0.619 (0.609 - 0.629)	0.626 (0.616 - 0.636)	0.632 (0.622 - 0.642)	0.619 (0.609 - 0.629)
11	0.619 (0.609 - 0.629)	0.626 (0.616 - 0.636)	0.631 (0.621 - 0.641)	0.619 (0.609 - 0.629)
12	0.618 (0.608 - 0.628)	0.626 (0.616 - 0.636)	0.631 (0.621 - 0.641)	0.618 (0.608 - 0.628)
13	0.618 (0.608 - 0.628)	0.626 (0.616 - 0.636)	0.630 (0.620 - 0.640)	0.618 (0.608 - 0.628)
14	0.618 (0.608 - 0.628)	0.626 (0.616 - 0.636)	0.629 (0.619 - 0.639)	0.618 (0.608 - 0.628)
15	0.617 (0.607 - 0.627)	0.626 (0.616 - 0.636)	0.629 (0.619 - 0.639)	0.618 (0.608 - 0.628)
16	0.617 (0.607 - 0.627)	0.626 (0.616 - 0.636)	0.628 (0.618 - 0.638)	0.618 (0.608 - 0.628)
17	0.617 (0.607 - 0.627)	0.626 (0.616 - 0.636)	0.627 (0.617 - 0.637)	0.617 (0.607 - 0.627)
18	0.617 (0.607 - 0.627)	0.626 (0.616 - 0.636)	0.627 (0.617 - 0.637)	0.617 (0.607 - 0.627)
19	0.617 (0.607 - 0.627)	0.626 (0.616 - 0.636)	0.626 (0.616 - 0.636)	0.617 (0.607 - 0.627)
20	0.617 (0.607 - 0.627)	0.626 (0.616 - 0.636)	0.626 (0.616 - 0.636)	0.617 (0.607 - 0.627)

[a] C-index=0.6338.

[b] C-index=0.6339.

[c] C-index=0.6337.

Table S4. Baseline characteristics of the validation lung cancer cohort

Characteristics	Overall (n=227)
Age, years	63.8 [57.4, 71.0][a]
Sex, male, n (%)	168 (74.0)
Smoking, yes, n (%)	138 (60.8)
Alcohol drinking, yes, n (%)	53 (23.3)
Clinical stage, n (%)	
I	16 (7.0)
II	43 (18.9)
III	38 (16.7)
IV	130 (57.3)
Anticancer therapies	
Radical surgery, n (%)	58 (25.6)
Curative chemotherapy, n (%)	86 (37.9)
Body mass index, kg/m^2	21.3 [18.1, 23.1]
Handgrip strength, kg	20.7 [16.2, 27.9]
Calf circumference, cm	32.0 [29.4, 34.2]
Triceps skinfold thickness, mm	11.0 [8.0, 16.0]
Neutrophil to lymphocyte ratio	3.4 [2.2, 6.1]
Prealbumin, mg/L	170.0 [111.5, 226.0]
Albumin, g/L	38.7 [35.4, 42.1]
Nutritional risk screening 2002 score, continuous	4.0 [2.0, 4.0]
PG-SGA score, continuous	7.0 [3.0, 13.0]
KPS score	70.0 [60.0, 80.0]
Global QOL score	50.0 [33.3, 50.0]
Length of hospital stay, days	11.0 [7.0, 20.5]

Abbreviations: PG-SGA, the Patient-Generated Subjective Global Assessment; KPS, the Karnofsky Performance Status; QOL, quality of life score by the European Organization for Research and Treatment of Cancer (EORTC) QLQ-C30 scale.

[a] Median [interquartile range], all such values.

Table S5. Multivariable models for the fat-age-inflammation (FAIN) index and overall survival in the validation cohort

	HR (95%CI), cases/events=227/126		
Models	Model 1[a]	Model 2[b]	Model 3[c]
FAIN, continuous	0.10 (0.04-0.23)	0.10 (0.04-0.27)	0.21 (0.06-0.76)
FAIN, per 1 SD (0.22)	0.59 (0.49-0.72)	0.60 (0.49-0.75)	0.70 (0.53-0.94)

Abbreviations: HR (95%CI), hazard ratio (95% confidence interval); SD, standard deviation; OS, optimal stratification.

[a] Model 1 is the unadjusted crude model.

[b] Model 2 is adjusted for the age at baseline (continuous).

[c] Model 3 is adjusted for the age at baseline (continuous), sex (reference = female), tumor stage (reference = I), radical surgery (reference = no), curative chemotherapy (reference = no), prealbumin (continuous), handgrip strength (continuous), the Nutritional Risk Screening 2002 (reference = less than 3), length of hospital stay (continuous), calf circumference (continuous), the Patient-Generated Subjective Global Assessment score (reference = 0 to 1), the Karnofsky Performance Status score (continuous) and the global quality of life score (continuous).
